# Translation, Reliability and Validity of the Greek Version of the 5-D Itch Scale in People With Chronic Kidney Disease Receiving Haemodialysis

**DOI:** 10.7759/cureus.78117

**Published:** 2025-01-28

**Authors:** Anastasia Liossatou, Aspasia Panagiotou, Dimitris Papageorgiou, Victoria Alikari, Georgia Gerogianni, Electra Lykiardopoulou, John D Stathoulis, Chara Tzavara, Marlyn J Mayo, Sofia Zyga

**Affiliations:** 1 Faculty of Health Sciences, Department of Nursing, University of Peloponnese, Tripoli, GRC; 2 Dialysis Unit, General Hospital of Kefalonia, Kefalonia, GRC; 3 Department of Nursing, University of West Attica, Athens, GRC; 4 Department of Digital Media and Communication, Ionian University, Kefalonia, GRC; 5 Biomedical Engineering, Sparta General Hospital, Sparta, GRC; 6 Biostatistics, National and Kapodistrian University of Athens, Medical School, Athens, GRC; 7 Department of Internal Medicine, University of Texas Southwestern Medical Center, Dallas, USA

**Keywords:** 5-d itch scale, chronic kidney disease, chronic kidney disease-associated pruritus, cross-cultural adaptation, haemodialysis, itching, nephrology nursing, reliability, translation, validity

## Abstract

Background: Chronic kidney disease-associated pruritus (CKD-aP) is a condition that causes significant distress to people undergoing haemodialysis (HD). It significantly impacts individuals' sleep habits, everyday activities, social interactions, mental health and overall quality of life. A number of tools have been developed to evaluate the severity of pruritus. The 5-D itch scale is one of the most widely used tools for assessing various aspects of pruritus. In Greece, there is no validated and culturally adapted tool to assess pruritus in individuals with CKD receiving HD. Hence, this study aimed to evaluate the reliability and validity of the Greek version of the 5-D itch scale.

Materials and methods: The English version of the 5-D itch scale was translated into the Greek language according to international guidelines and was cross-culturally adapted in people with CKD on HD (Ν = 120). The intraclass correlation coefficient was used to assess the reliability of the instrument after the test-retest procedure (n = 30). Confirmatory factor analysis was conducted in order to test how well the 5-D itch scale one-factor model fits the data. Internal consistency reliability was determined by the calculation of Cronbach’s alpha coefficient (n = 120). Convergent validity was tested through the correlation with the Kidney Disease Quality of Life Instrument Short-Form (KDQOL-SF) scale and the visual analogue scale. Statistical significance was set at p < 0.05. Analyses were conducted using IBM SPSS Statistics for Windows, version 26.0 (released 2019, IBM Corp., Armonk, NY).

Results: The sample consisted of 120 people with CKD receiving HD (60.0% males, n = 72), with a mean age of 65.7 years (SD ±13 years), of whom 80 individuals reported CKD-aP. Regarding confirmatory factor analysis, this solution had a good model fit (χ2/df = 1.93; root mean square error of approximation (RMSEA) = 0.03; comparative fit index (CFI) = 0.92; Tucker Lewis index (TLI) = 0.93 and standardized root mean square residual (SRMR) = 0.05). The intraclass correlation coefficients were significant (p < 0.001) in all items and over 0.85. Cronbach’s alpha for the 5-D itch scale total score was 0.75, indicating good reliability. The reliability index had minor changes after removing its items; thus, no item needed to be removed from the scale. The correlation of the 5-D itch scale with the KDQOL-SF (p = 0.002) indicates the good convergent validity of the scale.

Conclusion: The Greek version of the 5-D itch scale is an instrument with good reliability and validity, possessing the potential to be utilised by healthcare professionals for the purpose of the assessment of CKD-aP among Greek-speaking people undergoing HD.

## Introduction

Chronic kidney disease-associated pruritus (CKD-aP) is a clinical condition causing significant discomfort to the majority of people with CKD, frequently impacting individuals undergoing haemodialysis (HD). It has profound effects on individuals’ sleeping patterns, challenges in daily activities, social life, depression and overall quality of life [1,2]. The prevalence of CKD-aP has varied significantly among different countries, possibly due to the diversity in the studies' tools used for measuring pruritus and the participants' cultural or economic differences, with rates of 82% in China, 71% in the UK, 60% in Germany and 57% in Belgium [3].

The overall prevalence of pruritus in people with CKD on HD is 55%, as indicated by data from a recent meta-analysis of 42 international studies [4]. According to the Dialysis Outcomes and Practice Patterns Study (DOPPS) (phases 4-6), it was reported that 67% of people on HD had experienced pruritus, with 37% indicating moderate-to-severe CKD-aP [1]. There continues to be no consensus on the aetiology and pathophysiology, identification and treatment options of CKD-aP [5,6], despite its high prevalence. This is attributed to its diverse clinical presentation, which varies in severity and anatomical distribution.

Numerous studies indicate that CKD-aP may significantly affect individuals' quality of life (QoL) as increased severity of pruritus is associated with a decline in QoL [1,2,6] and could be as incapacitating as chronic pain [7] due to disturbances in the mood and sleep disorders and have exhibited with adjustment difficulties to their kidney condition and fatigue [1,4,5]. It was reported that individuals with severe pruritus exhibited a lower level of adherence to their medication and HD regimens [1]. Furthermore, individuals with chronic pruritus have demonstrated a higher incidence of hospitalisations related to cardiovascular complications or infections [1], a prolonged recuperation time from HD sessions [1,2,8], a higher morbidity and an increase in mortality [9]. However, CKD-aP is still considerably underestimated by healthcare providers, underreported by individuals and, as a result, undertreated by the healthcare team [1,3,9].

The evaluation of various dimensions of pruritus is imperative, given that it is one of the most prevalent and distressing symptoms among individuals on HD. Several instruments have been established to evaluate the severity of pruritus, including the 5-D itch scale [10], the visual analogue scale (VAS) [11], the Eppendorf Itch Questionnaire [12] and the Skindex-10 [13]. The 5-D itch scale, devised by Elman et al. (2010), is a prominent tool for evaluating a variety of itching dimensions and is extensively used among these instruments. The 5-D itch scale is a concise and comprehensible instrument for patients that encompasses all dimensions of pruritus. To the researchers’ knowledge, no validated tool exists in the Greek language for assessing pruritus in Greek-speaking people with CKD receiving HD. The Greek version of the 5-D itch scale is an essential instrument for the evaluation of Greek-speaking people with CKD experiencing pruritus while maintaining cultural and language relevance. Language is essential for accurately interpreting symptoms, emotions, or behaviours; a literal translation of psychological or medical scales might not capture the variations of meaning in various cultural contexts. Through the adaptation of the 5-D itch scale for Greek speakers, healthcare professionals can obtain more meaningful and accurate assessments, resulting in enhanced care for individuals with CKD-aP. Therefore, the aim of this study was to evaluate the psychometric properties (reliability, internal consistency and validity) of the Greek version of the 5-D itch scale (see Appendix A).

## Materials and methods

This is a cross-sectional, descriptive study carried out during March-July 2023 among people with CKD on HD (N = 120) who were randomly selected from four HD units (one from the Ionian Islands, The General Hospital of Kefalonia and three from the Peloponnese region, The General Hospital of Pyrgos, The General Hospital of Tripoli and The General Hospital of Molaoi) affiliated with the 6th Regional Health Authority of the Peloponnese and the Ionian Islands, Greece. The literature indicates that to assess the reliability and validity of the tools corresponding to each question in the questionnaire, a sample size of 10 participants is required [14]. Hence, the number of 120 participants is adequate, considering that the 5-D itch scale comprises five questions. The inclusion criteria for participants were a minimum of six months on HD, being over the age of 18 and possessing the capacity to read and write in the Greek language. The study precluded individuals with mental disorders and impaired eye vision, as this could have resulted in erroneous interpretations or inaccuracies in completing the scale. In addition, participants with hepatic complications and those with autoimmune diseases, including systemic lupus erythematous, were excluded from the study due to the overlapping clinical presentation that is similar to the pruritus associated with kidney involvement [15]. The researcher approached the participants either during their HD session or while they were awaiting entry to the HD unit prior to their treatment.

Instrument

The 5-D itch scale, established by Elman et al. in 2010, is a self-reported instrument that evaluates the intensity of pruritus and its effects on the QoL and can identify changes over time [10]. It contains five domains that assess the duration, intensity, direction, disability and distribution of pruritus. The 5-D itch scale scores vary from 5 (absence of pruritus) to 25 (maximum severity of pruritus). Among these five domains, three are single-item domains (duration, degree and direction) with values spanning from 1 to 5. The disability domain has four sub-items, and its score is determined by selecting the highest score from each of the four items. The concluding section addresses the distribution of pruritus across various body regions. Body parts numbered 0 to 2 yield a score of 1, those numbered 3 to 5 yield a score of 2, the total of body parts numbered 6 to 10 yields a score of 3, the total from body parts numbered 11 to 13 yields a score of 4, and the total from body parts numbered 14 to 16 yields a score of 5.

Translation process and cross-cultural adaptation of the Greek version of the 5-D itch scale

The process of translation and cross-cultural adaptation of the English version of the 5-D itch scale to the Greek-speaking people was carried out in compliance with the international guidelines [16] as follows:

Forward Translation

Two versions of the forward translation of the 5-D itch scale from English to Greek were developed by two bilingual translators whose mother tongue was Greek. One translator was an English literature teacher, and the other translator was a health professional from the field of nephrology.

Synthesis/Merging of the Two Translations From English to Greek

The review committee consisting of the two researchers compared the two versions of the translation and synthesised the first version of the 5-D itch scale in Greek.

Reverse Translation

Two versions of backward translations of the 5-D itch scale from Greek to English were developed by one teacher of English literature whose mother tongue was Greek and one health professional from the field of nephrology.

Synthesis/Merging of the Two Translations From Greek to English

The same review committee as described above compared the two versions of the translation and concluded to one version of the 5-D itch scale in English as the back-translated version.

Comparison of the Two Scales in Greek and English

The original English version of the 5-D itch scale (Appendix B) was compared with the back-translated one. There were no major discrepancies found in the comparison between the two texts. However, some minor differences were identified. In particular, an adjustment was made regarding the correct interpretation of the domain 'degree' to accurately convey the meaning of 'intensity', while the domain 'direction' was interpreted to signify 'a course along which something moves', specifically referring to 'evolution'. In addition, the domain 'disability' was defined as 'a reduction in the individual's ability to function', while the domain 'distribution' was expressed as 'the areas of appearance of itching'. Furthermore, consideration was given to the term employed at the scale that most accurately describes 'itch' or 'pruritus'' in the Greek language. Hence, in order to guarantee that the participants in the study could comprehend the word [knizmós]-[Κνησμός], which translates to 'pruritus' or 'itch' in Greek, it was decided to incorporate the term 'faγúra]-[φαγούρα]' into a parenthesis. Hence, the required adjustments were made to the Greek-translated text in relation to the two scales' conceptual, experiential, idiomatic and semantic equivalency. From this process, the semi-final version of the 5-D itch scale in the Greek language emerged.

Cross-Cultural Adaptation and Test of the Pre-final Version

The semi-final version of the Greek translation of the 5-D itch scale was distributed to a sample of 30 participants who were questioned regarding the relevance, comprehensibility, clarity and importance of the scale’s questions, as well as the ease and sufficiency of the response options. Any potential suggestions from the participants for improvement and modification would be taken into consideration to adapt and develop the final version of the 5-D itch scale in the Greek language (see Appendix).

Reliability

In order to assess the reliability of the scale, the test-retest method was used. An evaluation of test-retest reliability was conducted on 30 participants who completed the scale twice within a two-week period. According to the literature, a two-week interval is required to prevent participants from recalling their first answers, allowing for the assessment of the consistency of their opinions upon repetition [17]. Moreover, the internal consistency was tested via Cronbach’s alpha Index. To study the convergent validity of the scale, the following scales were used:

a) The Kidney Disease Quality of Life Short Form (KDQOL-SF) scale is a tool used to assess the health-related QoL (HRQOL) of individuals with CKD undergoing HD. The KDQOL-SF evaluates the QoL by integrating the kidney disease-specific KDQOL with the generic SF-36. Scores, converted to a 0-100 scale, indicate improved HRQOL with increased values. The SF-36 comprises eight subscales, such as physical functioning and mental health, which generate physical and mental summary scores. The KDQOL incorporates 11 subscales, encompassing the burden of kidney disease, cognitive function and patient satisfaction, generating a comprehensive assessment of kidney illness. The KDQOL-SF is multidimensional, concise and simple to use. This tool has been validated in the Greek language [18].

b) The VAS is a patient-reported outcome measure that is used to assess the severity of itch over a four-week period. It is structured as a horizontal line with indicators that range from 'no itchiness' (0 mm) on the far left to 'worst possible (or 'unbearable') itchiness' (100 mm) on the far right. The individual employs abstract thinking to interpret the severity of their itch into a horizontal line position that requires manual measurement of the mark with a ruler. The overall rating is between 0 and 10 [11].

Data analysis

Quantitative variables were presented as mean (standard deviation) or median (interquartile range). Categorical variables were represented as absolute and relative frequencies. The normality of quantitative variables was assessed using the Kolmogorov-Smirnov test. Spearman’s correlation coefficients (rho) were employed to explore the convergent validity of the 5-D itch scale through the correlation with the KDQOL-SF scale [18]. The intraclass correlation coefficient (ICC) was employed to evaluate the reliability of the questionnaire following the test-retest procedure. A confirmatory factor analysis (CFA) utilising the maximum likelihood estimation method was performed to evaluate the fit of the one-factor model of the 5-D itch scale to the data. The goodness-of-fit indices utilised include the Chi-square by degrees of freedom ratio (χ2/df), the Comparative Fit Index (CFI), the Tucker Lewis Index (TLI), the Root Mean Square Error of Approximation (RMSEA) and the Standardised Root Mean Square Residual (SRMR) [19]. According to the literature, these parameters are deemed adequate when the following criteria are met: χ2/df ≤ 2.0, CFI ≥ 0.90, TLI ≥ 0.90, RMSEA ≤ 0.05 and SRMR < 0.08 [19,20]. The calculation of Cronbach’s alpha coefficient and the item-total correlation coefficients was utilised to estimate internal consistency reliability. In order to assess and interpret internal consistency, reliability is classified as excellent (α ≥ 0.9), good (0.7 ≤ α < 0.9) and acceptable (0.6 ≤ α < 0.7). All reported p-values were analysed using a two-tailed approach. Statistical significance was established at p < 0.05, and analyses were performed utilising IBM SPSS Statistics for Windows, version 26.0 (released 2019, IBM Corp., Armonk, NY).

## Results

The sample consisted of 120 people with CKD receiving HD (60.0% males, n = 72), with a mean age of 65.7 years (SD ±13 years), of whom 80 individuals reported CKD-associated pruritus. The participants’ characteristics are presented in Τable 1. The majority of the participants had completed secondary education (55.8%, n = 67), were married (66.1%, n = 78), had children (70.0%, n = 84) and were pensioners (68.9%, n = 82). Moreover, 47.5% (n = 56) of the participants had a monthly income of 500-1,000 euros, and 52.5% (n = 63) were living in a rural area. Moreover, 35% (n = 42) of the sample were overweight, 21.7% (n = 26) were smokers and 20% (n = 24) were physically active. The mean time that individuals were on HD was 6.7 years (SD ±6.4) and their mean age at the time that they started was 58.4 years (SD ±16.2 years). Table 1 shows the sample characteristics.

**Table 1:** Sample characteristics

N = 120		N (%)
Gender	Females	48 (40)
	Males	72 (60)
Age, mean (SD)		65.7 (13)
Educational level	None	12 (10)
	Primary	29 (24.2)
	Secondary	67 (55.8)
	University	12 (10)
Family status	Unmarried	27 (22.9)
	Married	78 (66.1)
	Divorced	3 (2.5)
	Widowed	10 (8.5)
With children		84 (70)
Number of children, median (IQR)		2 (2 - 3)
Working status	Part-time employed	8 (6.7)
	Household	19 (16)
	Pensioner	82 (68.9)
	Unemployed	10 (8.4)
Monthly income (euro)	<500	29 (24.6)
	500-1000	56 (47.5)
	1001-1500	22 (18.6)
	1501-2000	10 (8.5)
	>2000	1 (0.8)
Place of residence	Urban	57 (47.5)
	Rural	63 (52.5)
ΒΜΙ (kg/m2), mean (SD)		26.2 (4.6)
ΒΜΙ	Normal	53 (44.2)
	Overweight	42 (35)
	Obese	25 (20.8)
Smoking		26 (21.7)
Daily number of cigarettes, mean (SD)		15.7 (8)
Physically active		24 (20)
Years in haemodialysis, mean (SD)		6.7 (6.4)
Vascular access	Fistula	73 (60.8)
	A-V Graft	4 (3.3)
	Central venous catheter	43 (35.8)
Age of haemodialysis onset, mean (SD)		58.4 (16.2)
Urea, mean (SD)		153.7 (36.9)
Creatinine, mean (SD)		9 (2)

Cross-cultural adaptation

Thirty individuals filled in the General Impression Instrument for the Greek version of the 5-D itch scale during its cross-cultural adaptation process. According to Beaton et al. (2000), 30 participants are required for this process [16]. The tool was generally perceived by the participants as easy to comprehend and use. In particular, 93% of the respondents reported that the scale's general impression was very good, 97% found the questions easily comprehensive, while 93% deemed the questions very important for their health status. In addition, 90% of the respondents identified no difficulties in completing the questionnaire (Table 2). Ultimately, no suggestions for improvement or modification of the final Greek version of the 5-D itch scale were provided by the participants.

**Table 2:** Results from the General Impression Instrument for the cross-cultural adaptation process of the 5-D itch scale

Items of General Impression Instrument	Responses’ options	Participants' response % (n = 30)
What is your overall impression of the scale?	Very good	93
	Good	7
Are the questions understandable?	Yes	97
	Difficult sometimes	3
Do you think the questions are relevant to your health condition?	Very important	93
	Not so important	7
Did you encounter any difficulties with the questionnaire responses’ options?	No	90
	Sometimes	10

Domains of the 5-D itch scale and their scoring

The domains of the 5-D itch scale and their scoring are described in Τable 3. In every domain, scores ranged from 1 to 5, delivering a cumulative score between 5 and 25. No score of 5 was recorded considering that pruritus was one of the participant’s entry requirements. The mean duration score was 1.17 (SD ±0.60), the mean degree score was 2.36 (SD ±0.80), and the mean direction score was 3.74 (SD ±0.65). The mean disability score was 2.19 (SD ±0.95), and the mean distribution score was 1.98 (SD ±1.13). The overall score ranged from 7 to 23, with the mean score being 11.43 (SD ±2.92). Three of these five domains are single-item domains (duration, degree and direction), with values extending from 1 to 5. The disability domain, on the other hand, is a multi-item domain that represents the impact of pruritus on daily activities, including sleep, leisure/social activities, housework/errands and work/school. To determine the disability domain score, the maximum score on any of the above-mentioned four items is considered. In the case of the present study sample, the participants' sleep was most significantly impacted by pruritus, with a sleep mean score of 2.13 (SD ±0.98) and 55.8% of participants reporting that they occasionally delay falling asleep. Consequently, the final score was adjusted to include the patient rating for the impact of pruritus on sleep (0-5) in order to evaluate the severity of the pruritus in these individuals. The impact of pruritus on the QoL was conveyed in terms of the rest domain items, which include social activities, housework/errands and work/school [10].

**Table 3:** Domains of 5-D itch scale and their scoring

N= 120		N	%
Duration (mean score = 1.17 ± 0.60)			
	Less than six hours/day	107	89.2
	Six to 12 hours/day	10	8.3
	12–18 hours/day	1	0.8
	18–23 hours/day	0	0.0
	All day	2	1.7
Degree (mean score = 2.36 ± 0.80)			
	Not present	6	5.0
	Mild	80	66.7
	Moderate	22	18.3
	Severe	9	7.5
	Unbearable	3	2.5
Direction (mean score = 3.74 ± 0.65)			
	Completely resolved	0	0.0
	Much better but still present	11	9.2
	Little bit better but still present	12	10.0
	Unchanged	94	78.3
	Getting worse	3	2.5
Disability (mean score = 2.19 ± 0.95) [Minimum=1, Maximum=5]			
Sleep (mean score = 2.13 ± 0.98)			
	Never affects sleep	28	23.3
	Occasionally delays falling asleep	67	55.8
	Frequently delays falling asleep	9	7.5
	Delays falling asleep and occasionally wakes me up at night	13	10.8
	Delays falling asleep and frequently wakes me up at night	3	2.5
Leisure/social (mean score = 1.08 ± 0.91)			
	Not applicable	39	32.5
	Never affect activity	39	32.5
	Rarely affects activity	36	30.0
	Occasionally affects activity	6	5.0
	Frequently affects activity	0	0.0
	Always affects activity	0	0.0
Housework/errands (mean score = 0.78 ± 0.65)			
	Not applicable	40	33.3
	Never affect activity	69	57.5
	Rarely affects activity	9	7.5
	Occasionally affects activity	2	1.7
	Frequently affects activity	0	0.0
	Always affects activity	0	0.0
Work/school (mean score = 0.67 ± 0.60)			
	Not applicable	48	40.0
	Never affect activity	64	53.3
	Rarely affects activity	8	6.7
	Occasionally affects activity	0	0.0
	Frequently affects activity	0	0.0
	Always affects activity	0	0.0
Distribution (mean score = 1.98 ± 1.13) (minimum = 1, maximum = 5)			
	Scoring Bin 1 (0-2 areas)	55	45.8
	Scoring Bin 2 (3-5 areas)	29	24.2
	Scoring Bin 3 (6-10 areas)	26	21.7
	Scoring Bin 4 (11-13 areas)	4	3.3
	Scoring Bin 5 (14-16 areas)	6	5.0
Total score (mean score = 11.43 ± 2.92)			
	5-10	69	57.5
	11-19	49	40.8
	20-25	2	1.7

**Figure 1:**
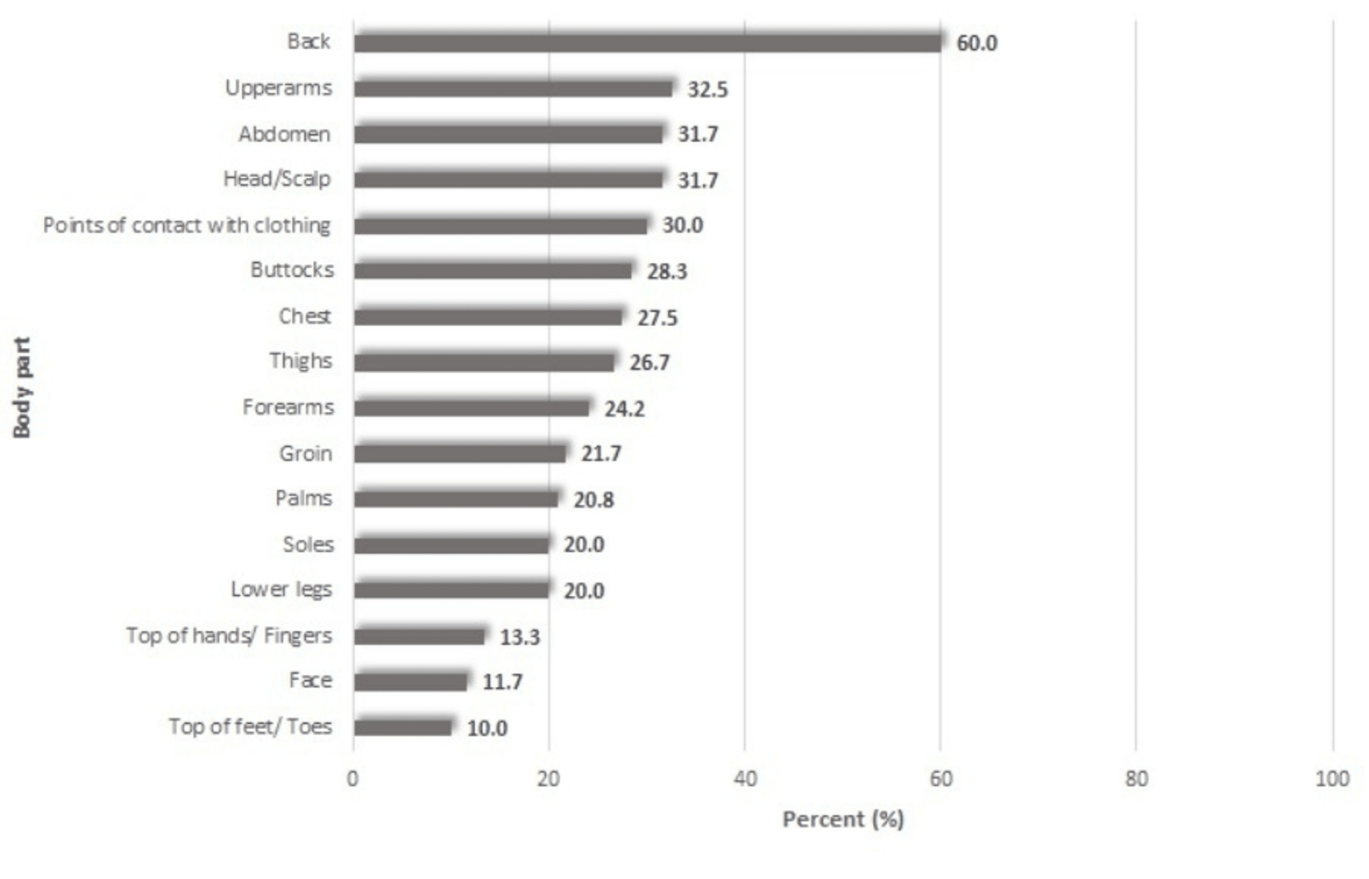
Percentages of itching in various body parts, by descending order

As far as the distribution of pruritus is concerned, which was the final domain in the 5-D itch scale, 16 body parts were included. Participants were given the opportunity to identify the body parts affected by itching. Considering the number of body parts, five scoring bins were established. The sum of the values 0-2¼ scores is 1, for 3-5¼ score is 2, for 6-10¼ score is 3, for 11-13¼ score is 4 and for 14-16¼ score is 5 [10]. The most frequent part of itching was reported to be the back (60%), followed by the upper arms (32.5%) and the abdomen (31.7%). In Figure 1, the distribution percentages of various body parts that participants experienced itching are illustrated.

CFA, test-retest results and internal consistency

CFA was performed to evaluate the one-factor solution of the original 5-D itch scale, revealing that this solution demonstrated a good model fit (χ2/df = 1.93; RMSEA = 0.03; CFI = 0.92; TLI = 0.93 and SRMR = 0.05). A CFA utilising the maximum likelihood estimation method was performed to evaluate the fit of the one-factor model of the 5-D itch scale to the data. The results of the test-retest are demonstrated in Table 4. The ICCs were significant (p < 0.001) in all items and over 0.85. Cronbach’s alpha for the 5-D itch scale total score was 0.75, indicating good internal consistency. The reliability index presented with minor changes after removing its items; thus, no item needs to be removed from the scale.

**Table 4:** Reliability of 5-D itch scale

	Test median	Re-test median	ICC	Corrected item total correlation	Cronbach's alpha if the item is deleted	P
Duration	1	1	0.97	0.46	0.73	<0.001
Degree	2	2	0.98	0.65	0.69	<0.001
Direction	4	3	0.85	0.08	0.78	<0.001
Disability						
• Sleep	2	2	0.96	0.60	0.70	<0.001
• Leisure/social	1	1	0.99	0.38	0.74	<0.001
• Housework/errands	1	2	0.87	0.63	0.71	<0.001
• Work/school	1	1	0.95	0.42	0.74	<0.001
Distribution	2	2	0.97	0.50	0.73	<0.001

All ICCs are significant at p < 0.001. The participants’ scores in the KDQOL-SF and VAS scales are presented in Table 5. The mean physical component score (PCS) was 34.46 (SD = 8.97) and the mean mental component score (MCS) was 42.9 (SD = 10.62). The median score in the VAS was 2 (IQR = 0-4).

**Table 5:** Participants’ scores in Kidney Disease Quality of Life Instrument Short-Form (KDQOL-SF) scales and visual analogue scale (VAS) IQR: interquartile range, PCS: physical component summary, MCS: mental component summary, VAS: visual analogue scale

	Mean (SD)	Median (IQR)
Physical functioning	44.45 (30.6)	50 (10-70)
Physical role functioning	17.71 (31.82)	0 (0-25)
Bodily pain	68.14 (24.78)	64 (52-100)
General health perceptions	34.68 (18.23)	35 (22-47)
Vitality	47.71 (20.96)	45 (30-65)
Social role functioning	59.27 (23.46)	50 (50-75)
Emotional role functioning	32.5 (45.62)	0 (0-100)
Mental health	63.17 (19.37)	66 (48-80)
PCS	34.46 (8.97)	35.33 (28.96-39.28)
MCS	42.9 (10.62)	41.13 (34.32-50.32)
Symptom problem list	82.61 (10.89)	85.42 (77.08-89.58)
Effects of kidney disease	53.38 (15.35)	53.87 (46.35-59.38)
Burden of kidney disease	45.83 (19.57)	43.75 (37.5-50)
Work status	12.5 (26.13)	0 (0-0)
Cognitive function	75.5 (14.52)	73.33 (70-86.67)
Quality of social interaction	73.17 (18.16)	73.33 (60-86.67)
Sexual function	64.64 (27.72)	75 (50-93.75)
Sleep	63.46 (14.17)	65 (55-72.5)
Social support	92.36 (14.96)	100 (83.33-100)
Dialysis staff encouragement	93.33 (12.01)	100 (87.5-100)
Patient satisfaction	89.86 (11.69)	100 (83.33-100)
VAS analogue scale, mean (SD)	2.56 (2.54)	2 (0-4)

Convergent validity

Spearman’s correlation coefficients (rho) were employed to investigate the convergent validity of the 5-D itch scale by examining its correlation with the KDQOL-SF Scale and VAS (Table 6). Increased levels of itching were significantly associated with a decline in QoL, according to most of the KDQOL-SF subscales (rho ranged from -0.18 (p = 0.050) to -0.41 (p < 0.001)). In addition, a greater score on the VAS was significantly associated with greater itching (rho = 0.67; p < 0.001).

**Table 6:** Spearman’s correlation coefficients between the 5-D itch scale and Kidney Disease Quality of Life Instrument Short-Form (KDQOL-SF) and visual analogue scale (VAS) IQR: interquartile range, PCS: physical component summary, MCS: mental component summary, VAS: visual analogue scale

	5-D itch scale	
	rho	P
Physical functioning	-0.25	0.006
Physical role functioning	-0.26	0.004
Bodily pain	-0.24	0.007
General health perceptions	-0.07	0.431
Vitality	-0.35	<0.001
Social role functioning	-0.35	<0.001
Emotional role functioning	-0.27	0.003
Mental health	-0.41	<0.001
PCS	-0.17	0.066
MCS	-0.38	<0.001
Symptom problem list	-0.38	<0.001
Effects of kidney disease	-0.18	0.050
Burden of kidney disease	-0.28	0.002
Work status	-0.22	0.015
Cognitive function	-0.05	0.555
Quality of social interaction	-0.25	0.006
Sexual function	-0.17	0.146
Sleep	-0.40	<0.001
Social support	0.02	0.822
Dialysis staff encouragement	-0.22	0.014
Patient satisfaction	-0.08	0.366
VAS	0.67	<0.001

## Discussion

This study aimed to evaluate the cultural adaptation, reliability and validity of the final translated Greek version of the 5-D itch scale, ensuring its suitability for its clinical application in Greek-speaking individuals with CKD undergoing HD. The translation process of the 5-D itch scale revealed no significant translational difficulties, and the translators along with the review committee easily reached a consensus over the final version of the Greek version of the 5-D itch scale. The translation of a text to another language may occasionally generate an illogical meaning when it is performed word-by-word. In the case of the Greek version of the 5-D itch scale, the minor differences identified between forward and backward translation led to the respective language modifications regarding the correct interpretation of the meaning of the scale's domains 'degree', 'direction', 'disability' and 'distribution'. Moreover, the researchers believe that including the term 'faγúra]-[φαγoύρα]', which means itch in parentheses at the beginning of the text's scale was essential to ensure that participants understood the word [knizmós]-[Kvησμός], which is the Greek word for Pruritus.

Furthermore, concerning the cross-cultural adaptation process of the 5-D itch scale, the majority of the participants reported that the scale's general impression was very good, found the questions easily comprehensive, deemed them very important for their health status and reported no difficulties in completing the questionnaire. Ultimately, no suggestions for improvement or modification of the final Greek version of the 5-D itch scale were provided by the participants.

The 5-D itch scale is a widely utilised PROM for pruritus, and according to the literature, it has been validated in multiple languages, including English (original), Arabic, Urdu (Pakistani), Malay, Japanese, Indonesian, Persian, Tai, Brazilian Portuguese and Spanish. Among the aforementioned versions of the 5-D itch scale, the Arabic, Urdu, Malay, Persian and Japanese editions have been utilised in individuals with CKD.

Internal consistency is commonly employed to assess the homogeneity of an instrument by analysing the relationships among several items within the same test. The internal consistency was evaluated using Cronbach’s alpha reliability coefficient. The Greek version of the 5-D itch scale demonstrated good internal consistency (Cronbach’s alpha = 0.75), identical to the Tai version (Cronbach’s alpha = 0.75) [21] and similar to the original English version (Cronbach’s alpha = 0.73) [10]. Compared with the Indonesian [22] (Cronbach’s alpha = 0.67), the Greek version showed higher internal consistency. By contrast, the Brazilian (Portuguese) [23] (Cronbach’s alpha = 0.80), Spanish [24] (Cronbach’s alpha = 0.83), Arabic [25] (Cronbach’s alpha = 0.85), Malay [26] (Cronbach’s alpha = 0.86), Urdu [27] (Cronbach’s alpha = 0.91) and Persian [28] (Cronbach’s alpha = 0.91) versions of the 5-D itch scale showed a higher internal consistency in comparison with the Greek version. Internal consistency was not reported in the Japanese version of the scale [29]; hence, it was not possible to compare it to the Greek version of the 5-D itch scale.

ICC values higher than 0.75 signify excellent agreement, those ranging from 0.60 to 0.74 denote good agreement, values between 0.40 and 0.59 reflect fair to moderate agreement, while values below 0.40 suggest poor agreement [30]. The ICC values for the test-retest reliability of the Greek version of the 5-D itch scale were significant (p < 0.001) in all items and over 0.85 (duration ICC = 0.97, degree ICC = 0.98, direction ICC = 0.85, disability: sleep ICC = 0.96, leisure/social ICC = 0.99, housework/errands ICC = 0.87, work/school ICC = 0.95), distribution ICC = 0.97), demonstrating good and excellent agreement, similar to the Arabic version (ICC = 0.85), Urdu version (ICC = 0.91), original version (ICC = 0.96) and the Persian version (ICC = 0.99).

The convergent validity of the 5-D itch scale was explored using Spearman’s correlation coefficients (rho) through its association with the KDQOL-SF scale and VAS. Higher itching was significantly correlated with poorer QoL according to most of the KDQOL-SF scales (rho ranged from -0.18 (p = 0.050) to -0.41 (p < 0.001)). In addition, a greater score in the VAS was significantly associated with higher itching (rho = 0.67; p < 0.001). The Greek version of the 5-D itch scale demonstrated a moderate correlation (rho = 0.67; p < 0.001) with the VAS, similar to the original English version of the scale (rho = 0.69) when correlated with the VAS.

A CFA employing the maximum likelihood estimation method was performed to evaluate the fit of the one-factor model of the 5-D itch scale to the data. The chi-square by degrees of freedom ratio (χ2/df), along with the CFI, TLI, RMSEA and SRMR, was utilised as goodness-of-fit indices [19]. These parameters were considered adequate, indicating that the solution demonstrated a good model fit (χ2/df = 1.93, RMSEA = 0.03, CFI = 0.92, TLI = 0.93 and SRMR = 0.05). A CFA was not performed on the other versions of the 5-D itch scale; therefore, it was not possible to compare the results of the CFA of the Greek version with the previous versions. Future research could concentrate on evaluating the CFA of the other versions of the 5-D itch scale to determine their validity and reliability across varied populations and clinical contexts.

Limitations

The researchers recognise several limitations. The use of convenience sampling for participants' recruitment in the study may have generated some bias, as it favours accessibility over representativeness, possibly resulting in the over- or under-representation of the target population within the specific geographical areas, demographics or with specific objectives. In addition, this study focused on people with CKD on HD, thus narrowing the generalisability of the findings to other populations with CKD. In particular, the results may not be entirely applicable to individuals with CKD receiving peritoneal dialysis or those with kidney failure without replacement therapy. Additional research is required to determine if the observed results are consistent across these varied patient populations.

Moreover, the scale's responsiveness was not examined; therefore, future studies are required to assess its responsiveness to treatment. Due to the insufficient availability of Greek-validated pruritus scales, convergent validity was performed employing the VAS and the KDQOL-SF. Future research may incorporate additional instruments to evaluate the validity of their tools. The Greek version of the 5-D itch scale could serve as an effective tool for clinicians to evaluate pruritus in individuals with CKD-aP in the Greek-speaking population. The instrument can be utilised in extensive studies examining different aetiologies of pruritus to evaluate itch severity and its impact on the QoL.

## Conclusions

The Greek version of the 5-D itch scale is an instrument with good reliability and validity for the evaluation of CKD-aP in the Greek-speaking population. Due to its strength qualities, the Greek version of the 5-D itch scale well captures the multi-dimensional aspects of pruritus, rendering it a valuable instrument for healthcare providers. The overall management care approaches of individuals receiving HD can be improved by using an objective, reliable and valid instrument.

The integration of the Greek version of the 5-D itch scale into clinical practice might optimise the QoL and management of individuals with CKD-aP. Therefore, the aforementioned instrument has the potential to be utilised by nurses and other healthcare professionals to evaluate CKD-aP in Greek-speaking individuals who are receiving HD in Greece and Cyprus. Moreover, the tool's applicability extends beyond Greece and Cyprus, reaching Greek-Cypriot communities in other countries such as the United States, Australia and Canada. Hence, the Greek version of the 5-D itch scale may serve as a valuable tool for non-Greek-speaking healthcare professionals delivering kidney care to Greek-speaking people with CKD undergoing HD. This ensures a comprehensive assessment of pruritus, enabling effective communication and appropriate management strategies across various clinical settings.

## Figures and Tables

**Figure 2:**
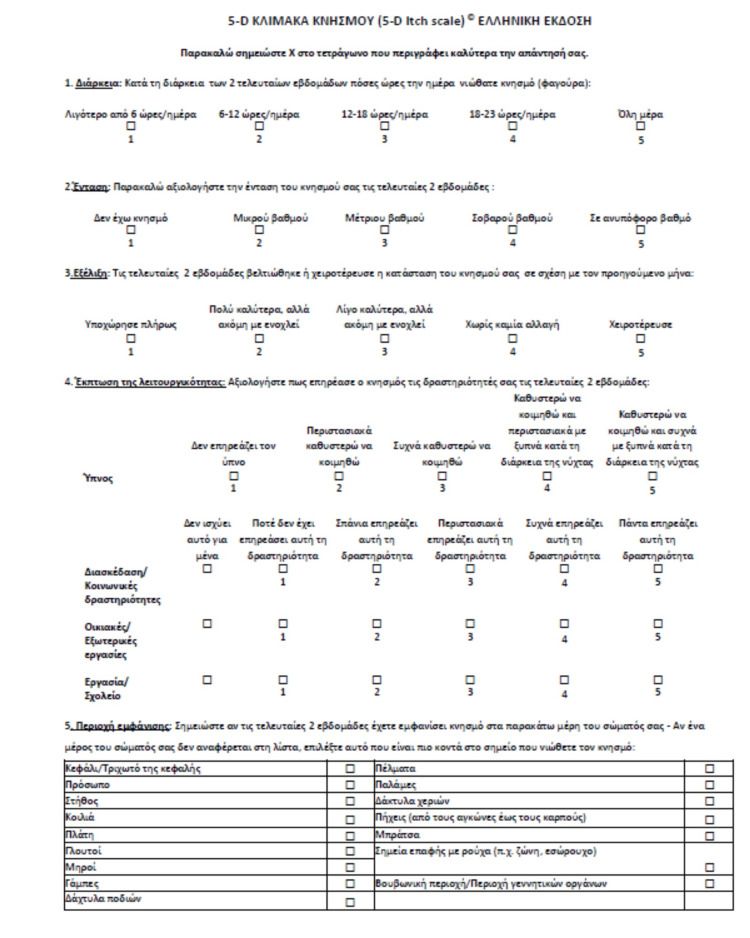
5-D Kλίμακα Κνησμού (5-D Itch scale) © Ελληνική έκδοση

**Figure 3:**
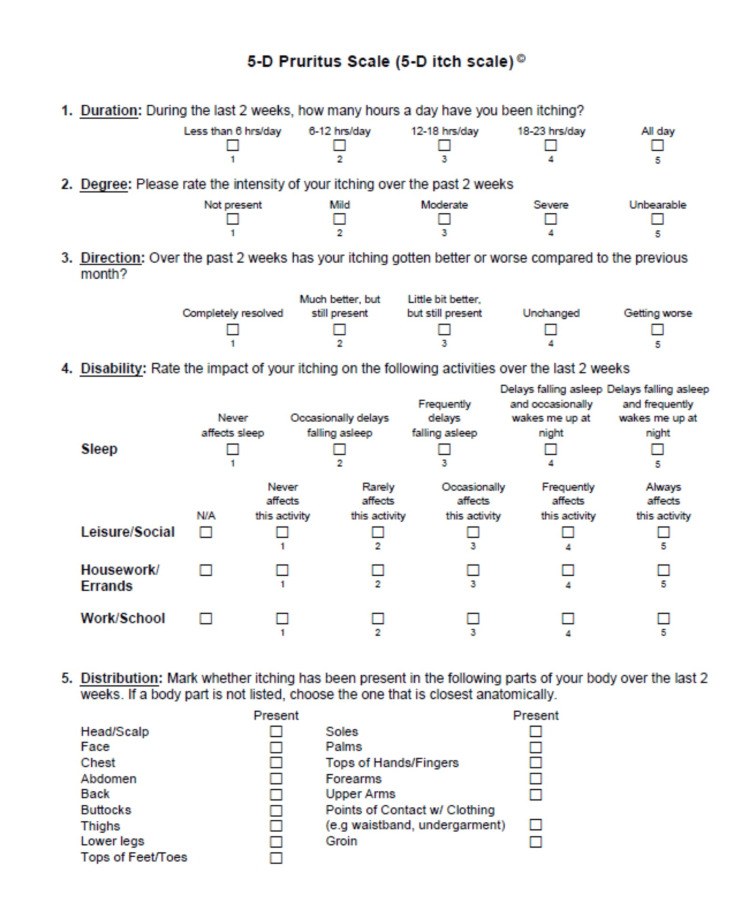
5-D Pruritus Scale (5-D itch scale) © English version

## Appendices

Appendix A

Appendix B
